# From Our Correspondents

**Published:** 1983-10

**Authors:** 


### From Our Correspondents

### Chair of Orthopaedics: Death of Foundation Professor

Many of your readers will have read of the death of Mr. David Fuller and his entire family in the helicopter crash off the Isles of Scilly on 16th July 1983.

He was appointed to the Foundation Chair of Orthopaedic Surgery on 6th September 1982 and was due to take up his appointment on 1st September 1983.

As Professor Williamson recorded in the January/April 1983 issue of this *Journal*, his Chair had been funded as a result of the successful efforts by the Trustees of the Bristol and South West of England Appeal for a Chair of Orthopaedic Surgery, to raise a sum of £450,000. The Appeal having been greatly honoured, and indeed aided, by H.R.H. The Prince of Wales, consenting to become its Patron.

After the announcement of the tragedy very many messages of sympathy had been received by members of the trust and one of the first was from our Patron, who also wrote at once to David Fuller's father. The Prince of Wales had asked to be kept informed of the steps being taken to fill the vacancy of the Chair.

The University Council at its meeting on 22nd July 1983 decided to proceed at once with the appointment of a successor to Professor Fuller. The post has already been advertised and it is hoped that an appointment will be made early in the New Year.

The death of David Fuller and his family was not only a personal tragedy of the first order but it also cut short a career of outstanding achievement and future promise.

His past achievements included research into drug-induced embryonic deformities, resulting in the award of the Degree of Master of Surgery (M.S.), the setting up of a specialist foot clinic in Oxford for the treatment of congenital deformities, working on the development committee and council of St. Luke's Nursing Home for the Disabled, a Christian Society, work for the parent teacher fund raising committee of his son's school, the appointment as Chairman of the Medical Executive Committee of the Nuffield Orthopaedic Centre, Oxford, and work for professional societies as their secretary and for the Medical Protection Society.

For the future he had thought out his first priorities on starting his work as Professor of Orthopaedic Surgery in Bristol. The plans were laid and in many cases the necessary funds had been obtained so that the work could start. These plans and hopes have by his death received a serious setback but there is every hope that a worthy successor will be appointed so that Orthopaedic Surgery in Bristol and the South West will receive the academic support which all desire.

The Memorial Service in the University Church of St. Mary the Virgin, Oxford was held on 17th September 1983. The church was filled to overflowing. The service was a moving and dignified one, ending with a prayer sung by the choir of his daughter's school which left many of the congregation very deeply moved.

M. P. MacCORMACK

### Computers in General Practice

In 1982 the Government made available over two million pounds for the 'Micros for GPs' scheme. This enabled 150 practices in the U.K. to install a microcomputer in their surgery for approximately half the full cost. The scheme was considerably oversubscribed and no Avon practice was selected as part of the scheme. Nonetheless at least three practices in Avon now have microcomputers working within their surgeries.

Lay people frequently assume that computers will do away with doctors – 'all you have to do is to programme a computer with all the alternative diagnoses and all the symptoms and signs and investigation results and the computer will do the rest'. Computers are not however used to aid diagnosis in general practice, in fact many of us question whether they ever will do this.

Their main task is to provide a general practitioner with quick access to his patients' records and to enable him to rapidly identify common factors within these records. It will enable him, for instance, to identify all patients who are receiving repeat medications of various kinds, to identify all patients who need their immunisations updating, and to remind him of routine investigations that need to be performed, such as haemoglobin checks in patients who have been anaemic or had certain surgical procedures performed.

General practitioners who consider they have a duty to follow-up patients with chronic conditions need some form of file to remind them who needs to be contacted. Many rely on card indexes, their memory or the memory of their patients but computers enable a quick and efficient search to be made. In our practice, for instance, we can search about 10,000 patient records in about an hour and can, for example, identify all patients on repeat digoxin medication. The advantage of computers over card indexes is simply one of convenience and speed: more alternatives can be held in a more accessible form.

How many practices will follow the present three is difficult to predict: it will depend on cost and benefit both of which will vary from general practitioner to general practitioner. Some will see the advantages, others will not. After all, there are still some general practitioners who do not have their routine referral letters typed!

M.J.W.

**Holistic and Alternative Medicine**

Recent articles in *The Times* on this subject have, in the opinion of most physicians, presented a biased and inaccurate view of the attitude of the medical profession on these subjects. There has never been a time when medical students, junior and senior doctors are more concerned with the total health of patients, meaning the effect of social, economic, emotional and other factors on their symptoms. I sometimes find student clerks know all about the family life of their patients and have forgotten to test the urine or examine the circulation in the legs! Then again on business ward rounds I find we spend an increasing amount of time discussing the social, housing and other personal problems, and put high technology aspects of biochemistry and radiology very much in the background, though the misinformed *Times* articles imply that doctors are obsessed with such complicated tests.

What are the reasons for the increased interest in alternative medicine by the public? I suggest that people have exaggerated expectations for their health, fuelled by television programmes which imply that new treatments are constantly being devised, whether they be operations for hypertension and arterial disease, mussel extracts or wonder drugs for rheumatoid disease, or 'therapies' for strokes, M.S. and spastic children. Patients with incurable diseases or psychosomatic complaints will readily try acupuncture, faith healing, herbal remedies, homeopathy, fancy diets, etc. One reason for their popularity is that many of the purveyors of alternative medicine can give more time to the patient than a doctor, and they impart enthusiasm which may well help morale. An additional explanation is the time-honoured fact that people appreciate something they have paid for themselves far more than drugs or treatment given by a state service at nominal charge.

My attitude to alternative medicine is that it is to be encouraged providing a qualified doctor has exhausted standard treatments, and providing there is no financial exploitation of ignorant victims. For example, I recently had a 22-year-old patient referred to me who had gone blind and emaciated whilst attending a 'healer'. She was a diabetic and subsequent treatment with Insulin restored her flesh, but not her sight. Then another patient of mine with M.S., following a programme on *Woman's Hour*, wrote off to London to be given an unpleasant diet and expensive bottles of vitamins which she took for a long time before realising their uselessness. I feel the medical profession must be prepared to study alternative treatments in a scientific way, preferably by a randomised trial, because *Digitalis* was, 200 years ago, only an old wives' tale.

H.G.M.

**Tribute to the Bands of the Salvation Army**

Although Frenchay Hospital is a hutted hospital with patients entirely at ground floor level, one of the great advantages of this type of building is that patients can wander freely in and out of the wards into the beautiful grounds that lie adjacent. This summer has been a bonus year for all of us, and patients have also made the most of the sunshine, with many of them able to enjoy the outdoor's life whether on foot, in wheelchairs or even in beds.

An interesting event occurs regularly out of doors on the morning of the second Sunday of every month, when the Staple Hill branch band of the Salvation Army marches up the lime-tree drive, and plays rousing music for half an hour from 10 a.m. until they depart to attend their own family service nearby. This gives immense pleasure to patients, many of whom wander out of the wards to watch. The band consists of between 40 and 45 bandsmen with an age range from 16 to 65 years, under the direction of Bandmaster Herbert Dunster. Major Hawken is the full-time minister in charge of the corps. The band stops three times up the drive, and plays hymn tunes, particularly those requested by patients on the wards. Some of the Army members go into the wards and chat to patients, particularly those seriously ill and at a low ebb, many of whom express their sincere gratitude and joy for the pleasure and comfort of their visit and the opportunity to hear a favourite hymn tune played.

It is of interest to learn that this event has been occurring for about 10 years with a short gap early on, and that the band also visits Cossham, Glenside and Meadowsweet hospitals on the other Sundays in the month. Band members attend entirely on a voluntary basis, and in fact provide their own uniforms and instruments. They pay a £5 subscription each year to belong to the band, and there is no charge for services. They even teach their own boys and girls from the age of 6 or 7 years who will then work their way up to qualify for membership of the senior band. In ordinary daily life, members would be found in any and every occupation such as insurance, electronic engineering, teaching, etc. Incidentally, another band from Horfield plays regularly at Southmead Hospital and one from the Citadel plays at the Royal Infirmary.

The Salvation Army does a wonderful job particularly amongst the poor, the lonely and the destitute. The pleasure and comfort that their bands must give, perhaps particularly to those sick and suffering, is not always recognised, but I am sure the patients, the nursing and medical staff would all like them to know how much their valuable work is appreciated, and express their gratitude to them.

J.L.G.T.

**How to Change the Balance of Staff to Meet a Changed Situation?**

For many years Southmead Hospital has been involved in training young pathologists. In the early years the two S.H.O.s worked mainly with Dr. Frank Lewis and many of these are now Consultants in Haematology. Amongst these are Dr. Ian Fraser who followed Dr. Lewis as our Haematologist at Southmead and has recently become Director of the South-West Regional Transfusion Centre based at Southmead.

Following Dr. Lewis's retirement, in the days of relative affluence in the N.H.S., we increased our complement of S.H.O.s to four and were able to offer a proper rotation through all four departments to provide an excellent basic training for young pathologists. It also provided an admirable year's training for potential clinicians in many fields of medicine and there was usually sufficient 'spare-time' to enable many of these non-pathologist S.H.O.s to obtain their M.R.C.P. during this period. In fact, at this time, the majority of the applicants came into this group and there were few candidates wishing to take up pathology. However, a few were deviated into one or other branch of pathology and amongst this group is Dr. Robert Slade, our new Haematologist. Appointment of these posts during this period was an easy task as there were rarely more suitable candidates than the number of jobs available.

Over the last two years, however, with the current excess of doctors for almost all types of post-registration jobs, we have now joined our surgical and medical colleagues in having an embarrassing number of excellent candidates, often Bristol trained, for our S.H.O. posts and their appointment is an invidious task. With the critical shortage of potential pathologists we now feel obliged to choose candidates who can persuade us that they are committed to a career in pathology. Sadly this means that we are now unable to offer future clinicians a year's experience in pathology.

This year we now face an additional factor when considering these posts – Finance. Due to the strict and increasing stringencies there has been a virtual freeze on any increase in technical staff for the laboratory for the past 18 months. This means that certain vital developments and improvements in the service we offer to clinicians have had to be curtailed or not even started. It is only natural that we are now casting a critical eye on our four S.H.O. posts. These are funded by Southmead District and if we are honest, apart from clinical commitments, they are almost a complete luxury as far as running the pathology service: indeed the work would be done quicker and possibly more efficiently without them. Although they provide a considerable teaching load, with a new face to be initiated into the basic elements of our four specialities every 3 months, we enjoy this challenge and would be sad to see them depart.

How can we solve this dilemma? If we are to continue to provide a service to our clinical colleagues, which must mean developing new techniques, must we now cease to train future pathologists? It is relevant that apart from similar training posts at the B.R.I. there are no other posts of this type throughout the South-West Region. Is it too much to hope that these essential training posts could be funded centrally and the money released be used, if only in part, to appoint the M.L.S.O.s we so urgently require? Maybe commonsense will prevail, but we are not too optimistic as this attribute is so often lacking in the planners at the D.H.S.S.

C.R.T.

**Ham Green Hospital**
**New Appointments**

We are delighted to welcome two new Consultants and their families to Ham Green.

Dr. John Harvey who replaced Dr. Robert Craig as Consultant Chest Physician comes to us from Bath where he has been Senior Registrar, rotating with the Bristol Royal Infirmary. He is not new to many of us as he has been a regular attender at our Area Chest Physicians Meetings for some time. He hopes to continue research started while at Bath and will, no doubt, develop new interests.

Dr. Iain Ferguson comes as a Consultant Neurologist to the Southmead District and replaces Dr. Francis Page. He was previously Consultant Lecturer in Neurology in Manchester and at Ham Green will continue the work started by Dr. Page in the Young Chronic Sick Unit (Orchard View, situated in the grounds of the hospital) and will also have some Neurology beds and will be available for consultation.

